# Epidemiology, classification, treatment and mortality of distal radius fractures in adults: an observational study of 23,394 fractures from the national Swedish fracture register

**DOI:** 10.1186/s12891-020-3097-8

**Published:** 2020-02-08

**Authors:** Johanna Rundgren, Alicja Bojan, Cecilia Mellstrand Navarro, Anders Enocson

**Affiliations:** 1 Department of Clinical Science and Education, Södersjukhuset, Karolinska Institute, Södersjukhuset, SE-118 83 Stockholm, Sweden; 2 Department of Orthopaedics, Sahlgrenska University Hospital Gothenburg/Mölndal, SE-431 80 Mölndal, Sweden; 3 Department of Molecular Medicine and Surgery, Karolinska Institute, Karolinska University Hospital, SE-171 76 Stockholm, Sweden

**Keywords:** Distal radius fracture, Epidemiology, Register study, Swedish fracture register, Surgical treatment, Fracture classification

## Abstract

**Background:**

Distal radius fractures are the most common of all fractures. Optimal treatment is still debated. Previous studies report substantial changes in treatment trends in recent decades. Few nation-wide studies on distal radius fracture epidemiology and treatment exist, none of which provide detailed data on patient and injury characteristics, fracture pattern and mortality. The aim of this study was to describe the epidemiology, fracture classification, current treatment regimens and mortality of distal radius fractures in adults within the context of a large national register study.

**Methods:**

We performed a descriptive study using prospectively registered data from the Swedish fracture register. Included were all non-pathological distal radius fractures registered between January 1st 2015 and December 31st 2017 in patients aged 18 years and above. Nominal variables were presented as proportions of all registered fractures.

**Results:**

A total of 23,394 distal radius fractures in 22,962 patients were identified. The mean age was 62.7 ± 17.6 years for all, 65.4 ± 16.0 for women and 53.6 ± 20.0 for men. A simple fall was the most common cause of injury (75%, *n* = 17,643/23,394). One third (33%, *n* = 7783/21,723) of all fractures occurred at the patients’ residence. 65% (*n* = 15,178/23,394) of all fractures were classified as extra-articular AO-23-A, 12% (*n* = 2770/23,394) as partially intra-articular AO-23-B and 23% (*n* = 5446/23,394) as intra-articular AO-23-C. The primary treatment was non-surgical for 74% (*n* = 17,358/23,369) and surgical for 26% (*n* = 6011/23,369) of all fractures. Only 18% of the AO-23-A fractures were treated surgically, compared to 48% of the AO-23-C fractures. The most frequently used surgical method was plate fixation (82%, *n* = 4954/5972), followed by pin/wire fixation (8.2%, *n* = 490/5972), external fixation (4.8%, *n* = 289/5972) and other methods (4.0%, *n* = 239/5972). The overall 30-day mortality was 0.4% (*n* = 98/23,394) and the 1-year mortality 2.9% (*n* = 679/23,394).

**Conclusion:**

This nation-wide observational study provides comprehensive data on the epidemiology, fracture classification and current treatment regimens of distal radius fractures in a western European setting. The most common patient was an eldery woman who sustained a distal radius fracture through a simple fall in her own residence, and whose fracture was extra-articluar and treated non-surgically.

## Background

Distal radius fractures (DRFs) are the most common orthopaedic fractures in the western world [1]. The distribution of DRFs in the general population is bimodal with incidence peaks in young men and in post-menopausal women [1–4]*.* DRFs in younger patients with good bone stock are most commonly associated with high-energy trauma [5], while low-energy trauma, e.g. fall from a standing position, is the most likely mechanism of injury in older patients due to underlying osteopenia/osteoporosis [6, 7]. Previous population-specific studies have reported both seasonal and weekly variations in the incidence of DRFs [3, 8–10].

Treatment of DRFs may range from elastic bandage to complex open surgery. The choice of treatment in each specific case is dependent on both fracture and patient characteristics. The optimal treatment for different types of DRFs and patient categories is still debated. Previous studies have high-lighted major changes in the treatment regimens of DRFs in the past two decades. Not only has the overall rate of surgically managed fractures increased, there has also been a significant increase in the use of internal plate fixation and a concomitant decrease in the use of percutaneous methods [4, 11].

The association between DRFs and a reduced bone mineral density in post-menopausal women is well established [6, 7]. Furthermore, a DRF in this patient group has been shown to increase the risk of subsequent osteoporotic fractures in the vertebrae, proximal humerus and proximal femur [12, 13]. The rising number of osteoporotic fractures in the western world due to greater life expectancy in combination with an increasingly active and demanding elderly population as well as more advanced and expensive treatment options pose a growing burden to the health care system [14–16].

Only a few nation-wide register studies of DRF epidemiology and treatment exist in the literature, and while they provide detailed description of incidence and changing treatment trends in recent years [4, 11, 17, 18]*,* they lack comprehensive data on fracture pattern, injury characteristics and mortality.

The Swedish fracture register (SFR) is a nation-wide register in which detailed data on orthopaedic fractures, patient characteristics, injury and treatment is reported prospectively. Previous publications from the register on specific fracture types include humeral, clavicle, tibial and proximal femoral fractures [19–22].

The aim of this study was to describe the epidemiology, fracture classification, injury characteristics, current treatment regimens and mortality in adult patients with DRFs, within the context of a large national register study.

## Methods

### Study population and data collection

Included in this study were all non-pathological distal radius fractures registered in the SFR between January 1st 2015 and December 31st 2017 in patients aged 18 years and above. We excluded all re-fractures, which we defined as a new fracture in the same wrist within 60 days.

The SFR is a national quality register on orthopaedic fractures and treatment, which started in 2011. Detailed data on patient and fracture chracteristics, injury mechanism as well as fracture treatment is registered prospectively at each affiliated department via a pre-specified digital form, usually by the treating doctor. Only patients with a permanent Swedish personal identity number and fractures that have occurred in Sweden are registered. In the SFR, fractures are classified according to the Arbeitsgemeinschaft für Osteosynthesefragen/Orthopaedic Trauma Association (AO/OTA) classification system [23]. Several studies have shown that this data has a high accuracy and validity [24–27]. The total number of orthopaedic departments in Sweden is 54. The proportion of departments affiliated to the SFR have increased gradually. At the start of this study, in January 2015, the number of affiliated departments was 27 and by the end of the study, in December 2017, it had risen to 45, which is estimated to cover more then 80% of the Swedish population. By the end of 2018 a total of more than 330,000 fractures had been registered in the SFR. Furthermore, the SFR is linked to the national Swedish death register, from which data on patient mortality was obtained.

### Variables

Epidemiological patient data (patient age and sex), injury data (injury location, cause and date), fracture data (fracture type, side, open/closed, trauma mechanism (high/low-energy), treatment data, as well as mortality data was retrieved from the register database.

Injury location was categorized as: at the patients’ residence or accommodation (including institutional housing), in a street/road, in a public place, or in an unspecified place. The cause of injury was categorized as: a simple fall (i.e. a fall in the same level), a fall from height, an unspecified fall, a traffic accident, or any other cause. The trauma mechanism was categorized as either high- or low-energy. There is no strict guideline for classification of the trauma energy level in the SFR and it is up to the registering doctor to distinguish. Fracture type was classified according to the AO/OTA classification system [23] and the International statistical classification of diseases and related health problems 10th revision (ICD-10) code system. Primary treatment type was divided into: primary non-surgical or primary surgical. The primary surgical treatment was further divided into: plate fixation, external fixation, pin/wire fixation, or any other method. Cases primarily treated non-surgically but with a secondary operation within 28 days were identified. Patient mortality was calculated and presented as 30-day and 1-year mortality.

### Statistics

Nominal variables are presented as proportions of all registered fractures, meaning the available number of inputs in the register excluding any missing values. Scale variables are presented as mean ± standard deviation (SD). Additional statistical testing of the variables was not performed because of the descriptive nature of the study. The statistical software used was IBM SPSS Statistics, Version 25 for Windows (SPSS Inc., Chicago, USA).

## Results

### Epidemiology

**Fig. 1:**
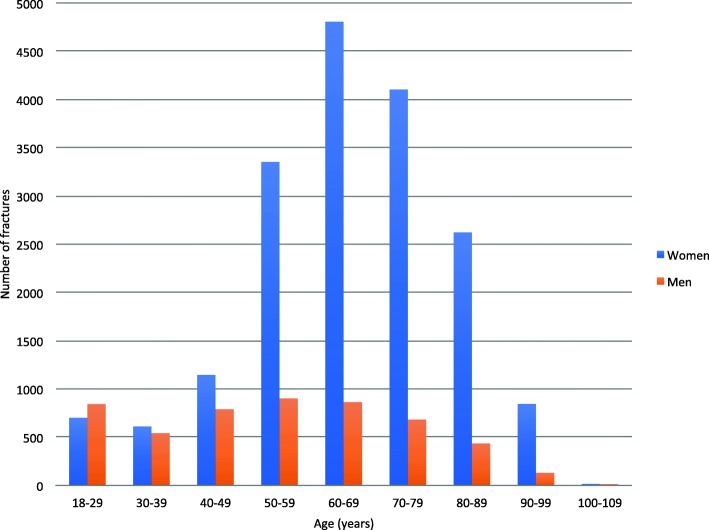
Distribution of distal radius fractures per age interval and sex

We identified a total of 23,394 DRFs in 22,962 patients. Of all patients, 1.6% (*n* = 373/22,962) had bilateral DRFs, 0.2% (*n* = 51/22,962) had a new DRF in the same wrist (i.e. occurring later then 60 days after the first registered DRF) and 0.02% (*n* = 4/22,962) had both bilateral and a new DRF in the same wrist during the study period. The majority of all DRFs (78%, *n* = 18,203/23,395) occurred in women. The overall mean ± SD age at the time of fracture was 62.7 ± 17.6 (range 18–104) years, while it was 65.4 ± 16.0 years for women and 53.6 ± 20.0 years for men. Data on the distribution of DRFs per age interval and sex is presented in Fig. 1.

### Injury characteristics

One third (33%, *n* = 7783/21,723) of the fractures occurred at the patients’ residence or accommodation, 13% (*n* = 3141/21,723) in a street/road, 8.6% (*n* = 2005/21,723) in a public place and 38% (*n* = 8794/21,723) in other unspecified places.

**Fig. 2:**
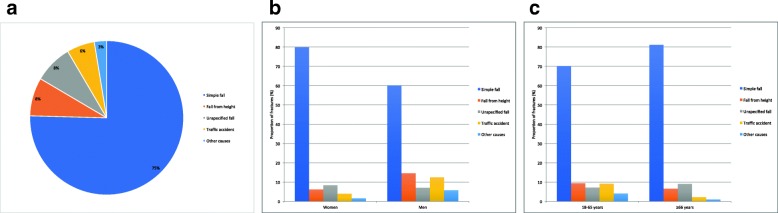
**a** Proportion of distal radius fractures (%) per injury cause. **b**. Proportion of distal radius fractures (%) for each injury cause in women and men respectively. **c**. Proportion of distal radius fractures (%) for each injury cause by age category (18–65 years or ≥66 years)

A simple fall was the most common cause of injury (75%, *n* = 17,643/23,394), while a fall from height represented 8% (*n* = 1880/23,394) of all DRFs, an unspecified fall 8.1% (*n* = 1892/23,394), a traffic accident 5.9% (*n* = 1372/23,394) and any other cause 2.6% (*n* = 607/23,394) (Fig. 2a). In Fig. 2b and c, data on the proportion of fractures by cause of injury and sex as well as by age category (18–65 or ≥66 years) is presented.

**Fig. 3:**
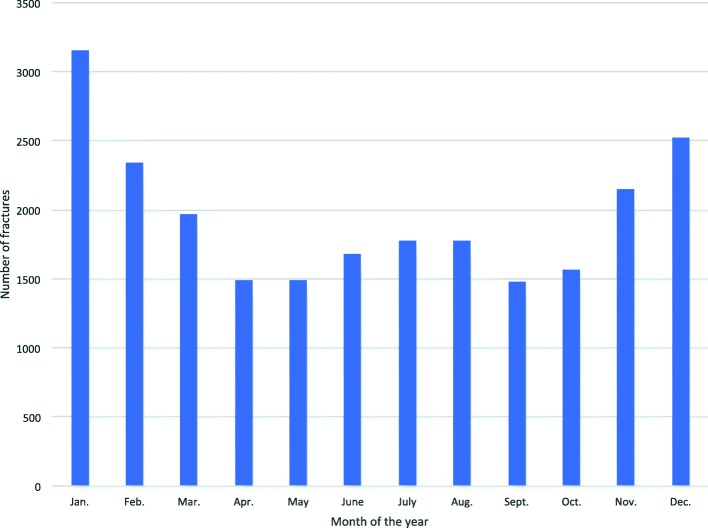
Distribution of distal radius fractures per month of the year

**Fig. 4:**
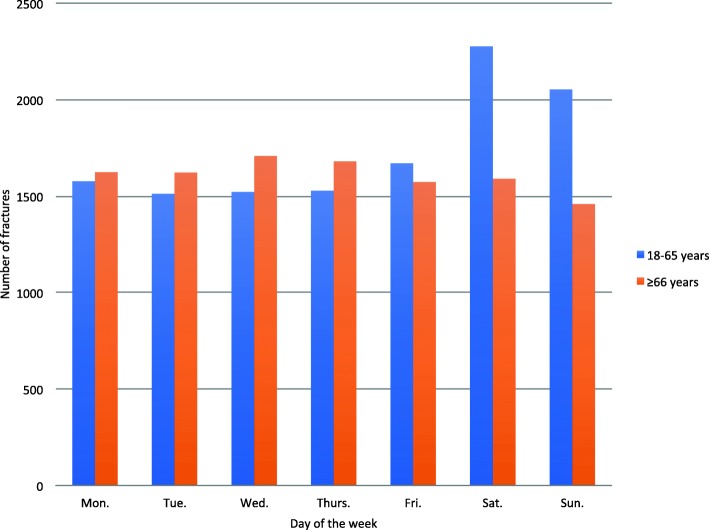
Distribution of distal radius fractures per day of the week by age category (18–65 years or ≥66 years)

DRFs were more common during the winter months (November through February) than the rest of the year (Fig. 3). Furthermore, DRFs were more frequent during the weekend compared to other weekdays for patients aged 18–65 years, while the distribution was similar throughout the week for patients aged ≥66 years. (Fig. 4).

### Fracture classification and characteristics

**Fig. 5:**
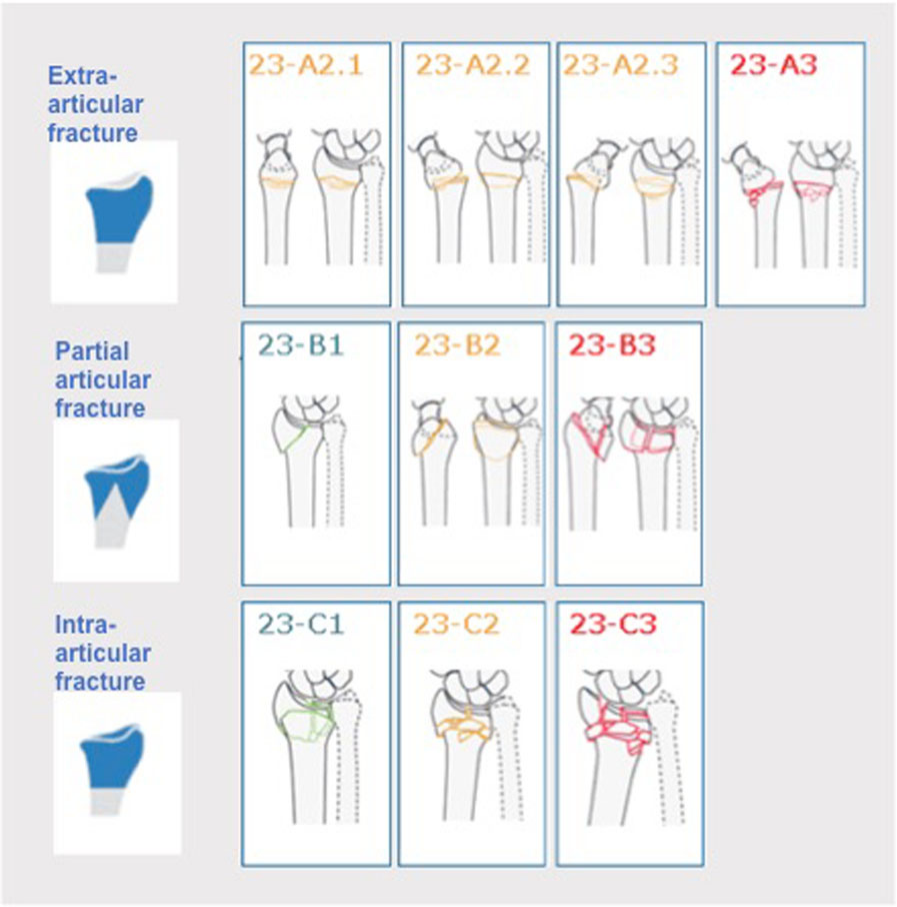
Schematic illustration of the AO/OTA classification system for distal radius fractures, as seen in the SFR

The majority of fractures were AO/OTA class 23-A2.1 (22%, *n* = 5126/23,394) or 23-A2.2 (31%, *n* = 7355/23,394). The AO/OTA classification system for DRFs with fracture types and groups is presented in Fig. 5. Detailed data on the distribution of DRFs by AO/OTA classification in relation to age, sex, open or closed fracture and trauma mechanism (high- or low-energy) is presented in Table 1.

**Table 1:** Distribution of distal radius fractures by AO/OTA classification in relation to age, sex, open/closed fracture and trauma mechanism (high/low-energy)

Fracture type	*n*= (%)	Mean ± SD age (years)	Women *n* = (%)	Open fracture *n* = (%)	High energy trauma *n* = (%)
23-A2.1	5126 (22)	58.7 ± 18.7	3851 (75)	2 (0.0)	167 (3.3)
23-A2.2	7355 (31)	66.1 ± 16.9	6244 (85)	41 (0.6)	181 (2.5)
23-A2.3	529 (2.3)	64.3 ± 17.1	435 (82)	12 (2.3)	31 (5.9)
23-A3	2168 (9.3)	65.8 ± 16.2	1838 (85)	80 (3.7)	94 (4.3)
Total 23-A	15,178 (65)	63.5 ± 17.8	12,368 (82)	135 (0.9)	473 (3.1)
23-B1	1377 (5.9)	56.0 ± 18.7	746 (54)	3 (0.2)	116 (8.4)
23-B2	638 (2.7)	61.8 ± 17.6	457 (72)	5 (0.8)	47 (7.4)
23-B3	755 (3.2)	61.3 ± 16.9	558 (74)	11 (1.5)	86 (11)
Total 23-B	2770 (12)	58.8 ± 18.2	1761 (64)	19 (0.7)	249 (9.0)
23-C1	2522 (11)	62.7 ± 16.4	1897 (75)	19 (0.8)	140 (5.6)
23-C2	1893 (8.1)	63.4 ± 16.4	1475 (78)	49 (2.6)	161 (8.5)
23-C3	1031 (4.4)	61.5 ± 16.6	702 (68)	67 (6.5)	179 (17)
Total 23-C	5446 (23)	62.7 ± 16.6	4074 (75)	135 (2.5)	480 (8.8)
All	23,394 (100)	62.7 ± 17.6	18,203 (78)	289 (1.2)	1202 (5.1)

The left wrist was slightly more commonly fractured (56%, *n* = 13,188/23,394). Only 1.2% (*n* = 289/23,394) of all DRFs were open fractures, and 5.1% (*n* = 1202/23,394) were registered as having a high-energy trauma mechanism.

According to the ICD-10 code system, fractures were classified as an isolated distal radius fracture (S52.5) in 92% (*n* = 21,534/23,394) and as a combined radius and ulna fracture (S52.6) in 2.0% (*n* = 1860/23,394) of the cases.

### Primary treatment

The primary treatment was non-surgical for 74% (*n* = 17,358/23,369) and surgical for 26% (*n* = 6011/23,369) of all DRFs. The mean age was 63.7 ± 18.3 years and 60.0 ± 15.1 years for patients with non-surgically and surgically treated fractures respectively. The proportion of women was similar in the non-surgical (78%, *n* = 13,479/17,358) and surgically treated groups (78%, *n* = 4699/6011). The mean time to surgery was 4.8 ± 3.7 (range 0–27) days.

**Fig. 6:**
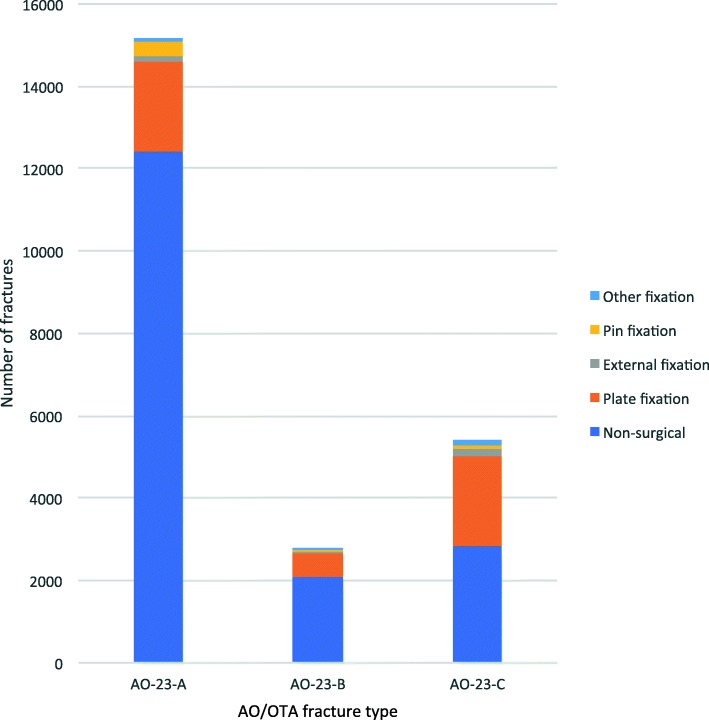
Distribution of distal radius fractures for each primary treatment type as well as surgical method by AO/OTA fracture type

The most frequently used primary surgical method was plate fixation (82%, *n* = 4954/5972), followed by pin/wire fixation (8.2%, *n* = 490/5972), external fixation (4.8%, *n* = 289/5972) and other methods (4.0%, *n* = 239/5972) (Fig. 6).

### Secondary surgical treatment

Of the DRFs that were treated non-surgically as primary treatment, 9.1% (*n* = 1586/17,358) underwent secondary surgical treatment. The mean ± SD time (days) to secondary surgical treatment was 11.2 ± 4.6 (range 1–28) days. The most commonly used method for secondary surgery was plate fixation (83%, *n* = 1319/1584), followed by pin/wire fixation (12%, *n* = 192/1584), external fixation (3.0%, *n* = 47/1584) and other methods (1.6%, *n* = 26/1584).

### Mortality

The overall 30-day mortality was 0.4% (*n* = 98/23,394) and the 1-year mortality was 2.9% (*n* = 679/23,394). The 30-day and 1-year mortality was similar for men (0.4%, *n* = 23/5191; 2.6%, *n* = 135/5191) and women (0.4%, *n* = 75/18,203; 3.0%, *n* = 544/18,203). When comparing mortality in relation to primary treatment, both the 30-day (0.5%, *n* = 92/17,358; 0.1%, *n* = 6/6011) and the 1-year (3.6%, *n* = 626/17,358; 0.9%, *n* = 52/6011) mortality was 4–5 times higher for patients treated non-surgically compared to surgically treated patients.

## Discussion

The main finding in this nation-wide descriptive study of DRF epidemiology, including 23,394 fractures registered in the SFR between the year of 2015 and 2017, was that the vast majority of DRFs occured in elderly women (≥50 years) as a result of a low-energy simple fall, often at the patients’ own residence. Between the age of 18 and 50, the distribution of DRFs was low and similar in both women and men. After the age of 50, however, there was a marked increase in the number of fractures in women, while the frequency of DRFs in men remained almost the same.

The substantial increase in the number of fractures in post-menopausal women can explain why more than three out of four DRFs occured in women in our study population. The ratio between women and men of 78:22 found in this study is in accordance with the result of a Swedish regional study [28], while it is sligthy higher than that reported in a Swedish national study; 75:25 [11] and substantially higher than what a British regional study reported; 68:38 [3].

### Fracture classification and characteristics

We found that the majority (65%) of all DRFs were extra-articular (AO-23-A), while 12% were partial articular (AO-23-B) and 23% complete intra-articular. The mean age was slightly lower in the AO-23-B and AO-23-C groups compared to AO-23-A group, while the proportion of men was higher and a high-energy trauma mechanism was more common.

Only 1.2% of all DRFs were open, which is in accordance with a previous regional register study of the Stockholm area, in which the proportion was 0.8% [29]. The proportion of open fractures varied between fracture groups, with the highest proportion (6.5%) in AO-23-C3 fractures. Furthermore, while the overall proportion of DRFs with a registered high-energy trauma mechanism was 5.1%, it was 17% in AO-23-C3 fractures.

We thus conclude that the AO/OTA DRF classes differed in some aspects of patient, injury and fracture characteristics.

### Primary treatment

In our study, 26% of all DRFs were treated surgically as the primary treatment. This number is higher than what was observed in a Swedish national register study of DRF epidemiology and treatment trends, in which the proportion of surgically treated fractures in 2005 was 16% and in 2010 20% [11]. We cautiously speculate that this may indicate a continued tendency to treat more DRFs surgically.

Of all DRFs treated surgically as the primary treatment, more than four out of five underwent internal plate fixation. This is in accordance with previous studies, which have reported that plate fixation has become the predominantly preferred surgical method in recent years in several countries [4, 11, 18, 29, 30].

While a majority (82%) of the AO-23-A fractures in the present study were treated non-surgically, almost half (48%) of the complete intra-articular DRFs (AO-23-C) were treated surgically. We speculate that this is likely due to an inherent instability and inacceptable joint surface incongruency in many intra-articular fractures, which often remains after closed reduction, and thus is a reason for surgery.

### Seasonal and weekly variation

We found a clear seasonal variation in the frequency of DRFs with an increased number of fractures during the winter months, i.e. November through February. Our results are in accordance with previous studies [3, 8–10, 31, 32]. It has been suggested that the increase in DRFs during winter is associated with more slippery walking conditions due to ice and snow [8, 10]. However, seasonality have been reported in countries and regions with mild winter climate as well, indicating that other factors also play a part [31, 32].

The number of DRFs did not vary between weekdays for patients aged 66 years or more, while there was a marked increase in fractures during the weekend (Saturday and Sunday) for patients between 18 and 65 years. Since the standard retirement age in Sweden is 65 years, we speculate that this is due to lifestyle differences between the two groups. Retired persons are likely to make less difference between the weekend and the rest of the week with regard to both in- and outdoor activies, while people who study or are employed tend to do outdoor and leisure activities during the weekend. Other studies have also reported weekly variations in DRF frequency or incidence with an increase in younger adults over the weekend [3].

### Mortality

The overall 1-year mortality of 2.9% in the present study is in accordance with results from both a Norweigan study [33] and a German study [34], in which an overall 1-year mortality rate of 3.4% and a 1.5 year mortality rate of 3.0% was reported respectively. However, our mortality rate is lower than the 1-year mortality of 6% reported in a Swedish regional study [35]. Our results regarding mortality in relation to primary treatment, showed a 4–5 times higher 30-day and 1-year mortality for non-surgically compared to surgically treated patients. We speculate that this may be explained by confounding by indication, i.e. that the patients treated non-surgically to a greater extent were older and more frail to start with, which may have been part of the reason why they received non-surgical treatment, and also may explain the higher mortality in that group.

### Strengths and limitations of the study

The large number of included fractures is a major strenght of this study. Furthermore, the SFR provides national and prospectively registered data, which reduces the risk of bias due to epidemiological and sociodemographical regional differences as well as varying local treatment traditions. Data on patient, fracture, injury and treatment characteristics is registered in the SFR in a pre-specified systematic way, which provides a detailed material on orthopaedic fractures and fracture treatment. To our knowledge, this study is uniqe both in size and detail with regard to the presented data on AO/OTA-classification of DRFs. Another strength of this study is that the data was collected in recent time and the study period only encompasses 3 years, which makes the results of this study both relevant and up-to-date.

An obvious limitation of this study is the lack of full national coverage of the SFR, although it improved during the study period so that by the end of the study the proportion of affiliated departments was more than 80% of all orthopaedic departments in Sweden. The incomplete national coverage is the reason why incidence rates could not be calculated. Another limitation is the lack of validation of specific DRF classification in the SFR.

## Conclusion

This observational nation-wide study provides comprehensive and up-to-date data on the epidemiology, classification, injury characteristics, current treatment regimens and mortality of distal radius fractures in a western European setting. The most common patient was an eldery woman who sustained a DRF through a simple fall in her own residence, and whose fracture was extra-articular and treated non-surgically.

## Data Availability

The datasets used and/or analyzed during the current study are available from the corresponding author on reasonable request.

## Abbreviations

AO: Arbeitsgemeinschaft für Osteosynthesefragen
DRF: Distal radius fracture
ICD-10: International statistical classification of diseases and related health problems 10th revision
OTA: Orthopaedic trauma association
SD: Standard deviation
SFR: Swedish fracture register
